# Digital Innovation, Data Analytics, and Supply Chain Resiliency: A Bibliometric-based Systematic Literature Review

**DOI:** 10.1007/s10479-022-04765-6

**Published:** 2022-05-19

**Authors:** Anas Iftikhar, Imran Ali, Ahmad Arslan, Shlomo Tarba

**Affiliations:** 1 International Lecturer in Logistics & Supply Chain Management, Lancaster University Management School, Lancaster University, Lancaster, United Kingdom; 2 Lecturer in Operations and Innovation Management, School of Business & Law, Central Queensland University, Rockhampton, Australia; 3 Oulu Business School, University of Oulu, Oulu, Finland; 4 Birmingham Business School, University of Birmingham, Birmingham, UK

**Keywords:** Digital innovation, Data analytics, Digital technology, Supply chain resilience, Bibliometrics

## Abstract

In recent times, the literature has seen considerable growth in research at the intersection of digital innovation, data analytics, and supply chain resilience. While the number of studies on the topic has been burgeoning, due to the absence of a comprehensive literature review, it remains unclear what aspects of the subject have already been investigated and what are the avenues for impactful future research. Integrating bibliometric analysis with a systematic review approach, this paper offers the review of 262 articles at the nexus of innovative technologies, data analytics, and supply chain resiliency. The analysis uncovers the critical research clusters, the evolution of research over time, knowledge trajectories and methodological development in the area. Our thorough analysis enriches contemporary knowledge on the subject by consolidating the dispersed literature on the significance of innovative technologies, data analytics and supply chain resilience thereby recognizing major research clusters or domains and fruitful paths for future research. The review also helps improve practitioners’ awareness of the recent research on the topic by recapping key findings of a large amount of literature in one place.

## Introduction

The innovative technologies of the 21st century, also called Industry 4.0 technologies, have now emerged as a new frontier to collect, and analyse real-time data and take more innovative decisions for sustained competitive advantage. Industry 4.0 or innovative technologies include the Internet of Things (IoT), blockchain technology, big data analytics, and artificial intelligence (Xu et al., 2018; Strozzi et al., 2017; Ali & Aboelmaged, 2022). These innovative technologies are integrated within a firm’s business environment where their operational processes, machines, and products constantly share data (Ivanov et al., 2019; Ali et al., 2021). Data analytics allow the firms to continuously identify the areas of improvement and thereby revolutionize their operations (Hoberg & Alicke, 2016). For instance, IoT connected devices collect and transmit data on supply and demand, inventory level, production planning and operational hiccups (Birkel & Hartmann, 2020; Kadadevaramth et al., 2020). Blockchain is capable to create visibility and transparency across the supply chain through the digitally distributed ledger, smart contracts, and multi-layered protection against financial threats (Cui et al., 2019; Lohmer et al., 2020). Big data analytics is found to be useful in enhancing data processing capacity and thereby responding to disruptive events (Dubey et al., 2019; Akter et al., 2020). The constantly collected data is evaluated to introduce the right products, improve product and service offerings, and share information throughout the network for performance optimization (Akter et al., 2020b). Also, valuable insights are gleaned from the data to compare the magnitude of the current disruptive event with the past events and devise a new strategy for quicker recovery. As such, technological innovation and data analytics has become an emerging topic from the perspective of supply chain resilience (SCRE).

The concept of SCRE mainly refers to preparing for, resisting and bouncing back to a more favorable equilibrium position in response to disruptions (Hendry et al., 2019). Generally, the extant literature suggests three phases—readiness, response and recovery—of supply chain resilience (Kamalahmadi & Parast, 2016; Ali & Golgeci, 2019). The readiness phase refers to the proactive deployment of resources to avoid possible disruptions (Kamalahmadi & Parast, 2016). The response phase comes into play when an incident materializes and thereby resists disruption to reduce the magnitude of losses. Whilst the recovery phase, which often follows the first two phases, refers to quickly recovering back from disruption and gaining a normal or even better than pre-disruptive stage (Ali et al., 2021). The recent global pandemic (COVID-19) and the complexity of global supply chains highlighted the use of innovative technologies to anticipate market changes and reduce the adverse impact (Xu et al., 2020). Consequently, we have observed the mounting research on the application of innovative technologies in managing disruptions and resilience in recent years (Hosseini & Ivanov, 2020; Ralston & Blackhurst, 2020; Zouari et al., 2021; Belhadi et al., 2021). Given that SCRE research is still nascent and evolving (Ali and Golgeci, 2019; Zouari et al., 2021), it is indispensable to map the emerging literature at the intersection of digital innovation, data analytics, and SCRE.

Prior studies offer some insightful literature reviews; however, their focus remains on SCRE alone. For instance, Ali et al., (2017) analysed the essential elements and managerial practices at different phases of supply chain resilience; Ali & Gölgeci (2019) identified numerous drivers, barriers, theories, moderators and mediators to build supply chain resilience through literature review and co-occurrence analysis; Xu et al., (2020) presented the development path in the area of supply chain disruptions through bibliometric analysis; Iftikhar et al., (2021) conducted a quantitative meta-analytic review on the antecedents and outcomes of supply chain resilience and firm performance. Further, the extant literature discusses literature reviews at the intersection of, for example, big data, and artificial intelligence in the maritime industry (Munim et al., 2020), big data and dynamic SC capabilities (Rialti et al., 2019; Mishra et al., 2018), big data and sustainable SC under big data (Zhang et al., 2020). As such, the existing literature reviews missed the opportunity to offer insights into the nexus of innovative technologies, data analytics, and SCRE. The disruptive occurrences, for instance, the Suez Canal blockage and natural disasters due to climate change (Ali and Golgeci, 2021) could cause failure in the manufacturing of numerous products used in everyday production, and delays in cargo shipments that rippled across the network (Iftikhar et al., 2022). The advancement in innovative technologies enables SC operations to continue by mitigating the disruptive impacts of the uncertain environment (i.e., pandemic) (Zhang et al., 2020). Researchers have argued that by utilizing the innovative technologies enabled data analytics, firms would be able to confront the complexity and dynamism in the external environment (Arslan et al., 2021), and hence improve resilience and robustness (Iftikhar et al., 2022; Ali & Govindan, 2021). Further, the resilience concept is multidisciplinary that necessitates a multifaceted approach (Iftikhar et al., 2021), the extant literature lacks reviewing the potential of innovative technologies enabled data analytics to build resilience across the supply networks. Therefore, a systematic literature review is indispensable in consolidating a scattered knowledge base and identifying current research trends and opportunities for impactful future research on an emerging topic.

In the extant literature, four main types of literature review approaches have been proposed (Paul & Criado, 2020). The first is referred to as a domain-based review, which allows literature review via computational methods, resulting in unique trends and themes in a particular field (Rialp et al., 2019; Paul & Criado, 2020). The second kind of review is classified as a theory-based review—analyzing the role of a particular theory in a specific area of interest or field (Rindfleisch & Heide, 1997; Colquitt & Zapata-Phelan, 2007). The third type includes the method-based review, which synthesizes the research studies based on a particular methodology – either quantitative or qualitative (Ding et al., 2018; Ji et al., 2019). The fourth commonly used approach is the meta-analytic reviews, which help quantitatively synthesize the existing empirical studies mainly through correlations of the underlying constructs (Ataseven & Nair, 2017; Iftikhar et al., 2021).

Despite the growing popularity of bibliometric analysis and systematic literature, there is an ongoing debate on the benefits of these two approaches. The proponent of bibliometric review underlines its ability to automatically generate research themes that have less bias compared to manually generated themes with systematic literature review (van Eck & Waltman 2010; Ali et al., 2021; Golgeci et al., 2022). Conversely, the partisans of systematic literature review argue that bibliometric studies are more focused on counting articles, the number of clusters, and contributing authors, thereby missing the opportunity to map the current state of the art and knowledge trajectories (Wong, 2021). Without indulging in the current debate, this study undertakes a more holistic perspective by combining the benefits of both bibliometric analysis and a systematic literature review. In doing so, we aim to offer a more reliable and biased free analysis that quantitatively generates the key research clusters or themes (bibliometric analysis) and thereby reviews the current state-of-the-art and the valuable avenues for future research in each cluster (systematic literature review). Formulation of the research question is indispensable to conducting a literature review in a more structured and logical order (Ali and Aboelmaged, 2021). As such, we derive the following three research questions for our review:

1. What are the key clusters of research and recent development in each cluster at the nexus of innovative technologies, data analytics and SCRE?
2. How has research on the topic evolved?
3. What is the methodological development/classification of the field?
4. What are the opportunities for more impactful research at the intersection of innovative technologies and SCRE?

The remaining paper is structured as follows. The second section discusses the literature review methodology of this study. The third section presents the findings of the bibliometrics analysis, along with the evolution of the research topic, knowledge trajectories, and classification of research methods. The fourth section outlines the discussion and future research directions. The paper is concluded with the study’s limitation in the fifth section.

## Review methodology

Consistent with the research objectives, this study combines bibliometric analysis with a systematic literature review. First, with the aid of visual tools (i.e., VOSviewer software), bibliometric analysis is employed to generate key research clusters based on the keyword’s association strength. While bibliometric analysis produces research clusters quantitatively and objectively, it is unable to describe the recent development or gap in each cluster. To overcome this shortcoming, we integrated bibliometric analysis with a systematic review approach where the articles related to each cluster were carefully reviewed by the authors of this study. Such a combination of two approaches thus helped our review to not only retrieve key research clusters in an objective and biased free manner but also discuss the recent research trajectories and opportunities for further research in each cluster. The past literature reviews show that the combination of these techniques provides more reliable findings (e.g., Ali and Golgeci, 2019). To follow the systematic review guidelines, we established the eligibility criteria (inclusion and exclusion criteria) for the present study (see Table 1). The data was collected from Scopus, which is a large source of scholarly publications in the field of business and management. To retrieve the maximum number of related articles, a comprehensive set of keywords with Boolean connectors was developed. In the extant literature, we couldn’t find a universal definition or agreement on what constitutes Industry 4.0. Different scholars suggested various technologies that can be categorized as Industry 4.0. As such, the keywords developed are motivated by some widely cited past studies on Industry 4.0 technologies (Xu et al., 2018; Strozzi et al., 2017; Tang & Veelenturf, 2019; Ivanov et al., 2019; Witkowski, 2017).

Our set of keywords included: “Supply Chain”, “Resilien*”, “Robust*”, “Disruption*” along with “innovative technologies*”, “Predictive analytics”, “Big Data”, “Industry 4.0”, “Big Data Analytics”, “blockchain”, “internet of things”, “IoT”, “Additive Manufacturing”, “3D Printing”, “RFID”, “Cloud computing”, “cyber-physical system*”, Sensors, “smart factory”, “advanced robotics”, “artificial intelligence”, “drones”, “Supply chain analytics”, “digital technologies*”, “digitization”, “digitalization”.

The initial search was conducted on 4th January 2021, which produced 589 articles in the English language within the subject areas of engineering, business management, science technology, operations research, operations management, transportation, and computer science. The research articles were searched starting from the year 2003, as the first publication on the SCRE topic appeared (Rice & Caniato, 2003). After retrieving the data, the first two authors of this study reviewed the dataset and found 262 relevant articles by looking at the titles, abstracts, and keywords. Given the nascency of the topic and to gain a comprehensive insight, we did not exclude conference papers, reviews, and book chapters. Overall, 327 were removed after evaluating the dataset according to the exclusion criteria mentioned in Table 1.

**Table 1:** Exclusion and Inclusion criteria

Criteria	Description
**Exclusion**	
A	Any article which is not categorized as a peer review article (editorial, or industry report).
B	Any article which is not written in the English language.
C	Any article not written at the intersection of Industry 4.0 technologies and supply chain resilience/disruptions.
D	Any article without full text.
**Inclusion**	
A	Any article which is peer-reviewed and is formally accepted for publication (forthcoming, ahead of print, or in press variants).
B	Any article belonging to any research method (empirical, case study, modelling, analytical, review).
C	Industry 4.0 enabled technology has to be a core concept of the article.
D	Articles must have discussed the concept of supply chain disruptions, resilience, or robustness along with Industry 4.0 enabled technology.

Adapted from Ghobakhloo et al. (2020)

After all inclusions and exclusions, we found a sample of 262 articles published between 2008 and 2021. The articles were exported to VOSviewer software for the bibliometric analysis. We used the “co-occurrence” function of the VOSviewer to generate key clusters of frequently occurring keywords. These clusters are generated based on the association strength of the keywords (Ali et al. 2021). Further, these clusters place the same characteristics of articles together. We have used the following equation to calculate the association strength:

1 $$ ASij = \frac{C_{ij}}{{w_i}{w_j}}$$

Where, c_ij_ denotes “the number of co-occurrences of items i and j and where w_i_ and w_j_ denote either the total number of occurrences of items i and j or the total number of co-occurrences of these items. It can be shown that the similarity between items i and j calculated using (1) is proportional to the ratio between, on the one hand, the observed number of co-occurrences of i and j and, on the other hand, the expected number of co-occurrences of i and j under the assumption that co-occurrences of i and j are statistically independent” (van Eck & Waltman 2010, 531).

The software helps in searching the frequently co-occurring keywords from the available data set extracted from SCOPUS and places them into different colors of clusters as per similar keywords. Based on similar keywords, each cluster represents closely related themes (Ali & Gölgeci, 2019). Also, the set of studies in these clusters pertains to a similar line of argument or themes.

**Fig. 1:**
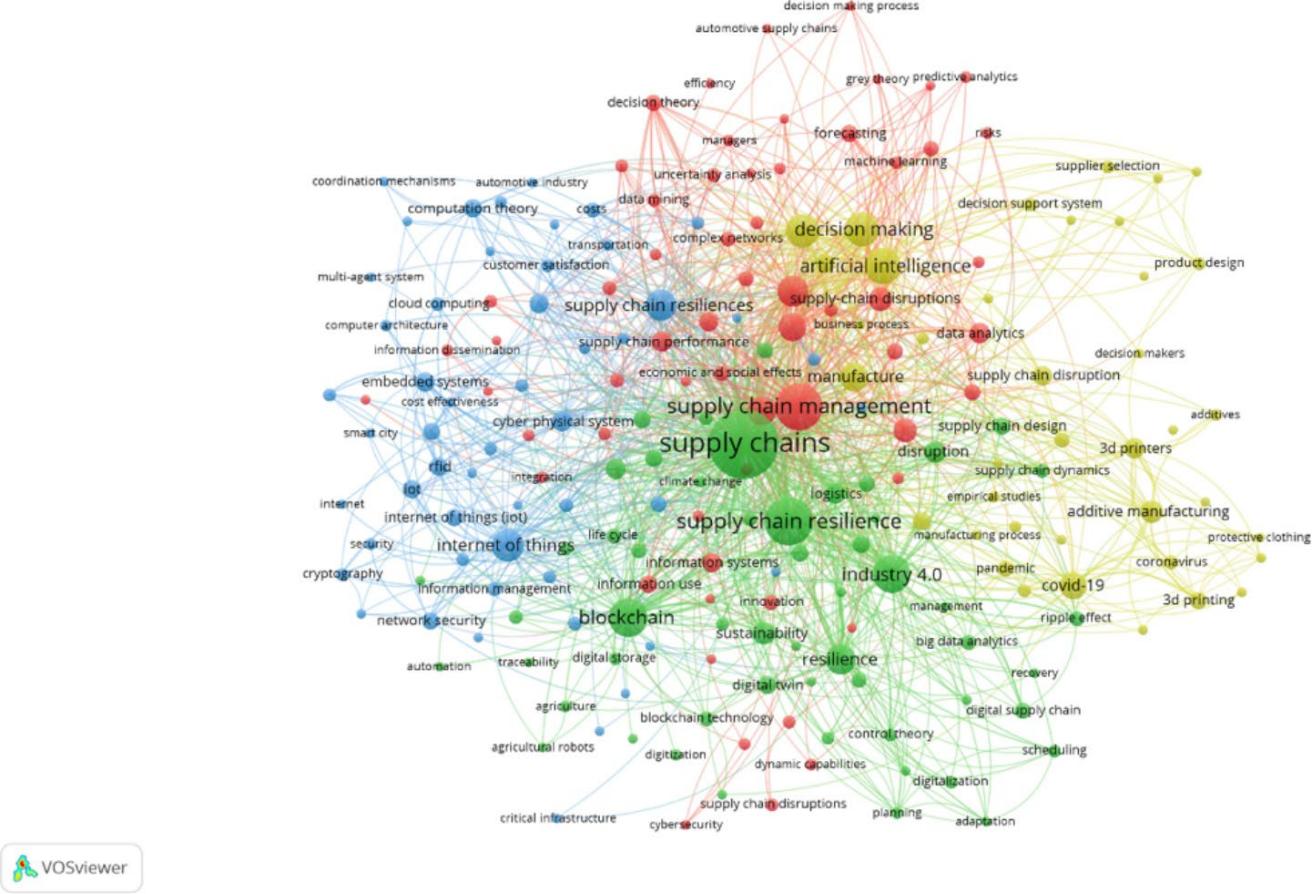
Clusters retrieved through VOSviewer analysis of 262 articles

Overall, the “co-occurrence” produced 4 different clusters (see Fig. 1) of research at the nexus of innovative technologies, data analytics and SCRE. The subsequent section discusses the major findings under each cluster.

## Findings of bibliometric analysis and systemic review

### Cluster 1: IoT and SCRE

Cluster 1 is the blue color cluster. The main keywords in this cluster include internet of things, SCRE, IoT, coordination mechanism, traceability, RFID, etc. Looking at the literature associated with these keywords, we classified this cluster as ‘IoT and SCRE’. The following section maps the key literature around cluster 1.

Exploring the nexus between IoT and SCRE, Al-Talib et al., (2020) and Cui (2015) argued that IoT technologies collect and transmit data to enhance visibility, flexibility, and collaboration, which are key precursors of the supply chain resiliency (Hohenstein et al., 2015). To protect the supply chains from highly impactful disruptions, Nah and Siau (2020) identified that the data collected through IoT technologies could be utilized to predict the sudden spikes in the demand and adapt and respond to the rapid market changes timely. This means that the implementation of IoT enabled innovative technologies will place firms in a much better position to anticipate disruptions, prepare themselves beforehand, reduce disruptive impact and assure uninterrupted supplies. Ivanov et al., (2019), Chen et al., (2020), and Park et al., (2020) further added that IoT supported cyber-physical systems could be integrated into the supply chain risk related data analytics, which is useful to navigate crises and help the firms in adapting to sudden changes (Kozyrkov, 2020). These authors also examined supply chain data analytics systems, concluding that big data analytics will be the primary source of competitive advantage in the future. This argument has also been supported by Gajek et al., (2020), Gelenbe et al., (2020), and Martin (2020) in that IoT will have a substantial influence on the competitiveness of the manufacturing sectors and, as a result, organisations should establish effective gateways for digital innovation. Going further, Birkel & Hartmann (2020) confound how the application of IoT in manufacturing companies can enhance transparency and risk knowledge to improve resilience performance. Similarly, Kadadevaramth et al., (2020) mentioned that IoT enables data analytics and resilience by providing access to real-time data through information sharing, collaboration, and pre-programmed responses. Hence, in today’s hypercompetitive and disruptive environment adoption of these highly sophisticated innovative technologies has become inevitable (Cui et al., 2019).

In increasingly complex SCs, with more stakeholders involved, IoT helps in designing intelligent and resilient SCs by creating real-time information sharing, visibility, and stronger collaboration among the business partners (Ali et al., 2021; Rejeb et al., 2020). Additionally, IoT also facilitates SCs in enhancing information transparency by strengthening the integration of information systems which then improves the firm’s data processing capabilities (Cui et al., 2019; Al-Talib et al., 2020). The beneficial effects of IoT are achieved through its key approaches, for instance, radio frequency identification (RFID), track and trace systems, wireless sensors, and coordination mechanisms, among others Yan et al., 2020; Burmester et al., 2017; Bemthuis et al., 2019). These elements are considered essential antecedents to improving the resiliency in SCs (Dubey et al., 2019; Ali & Golgeci, 2019).

**Fig. 2:**
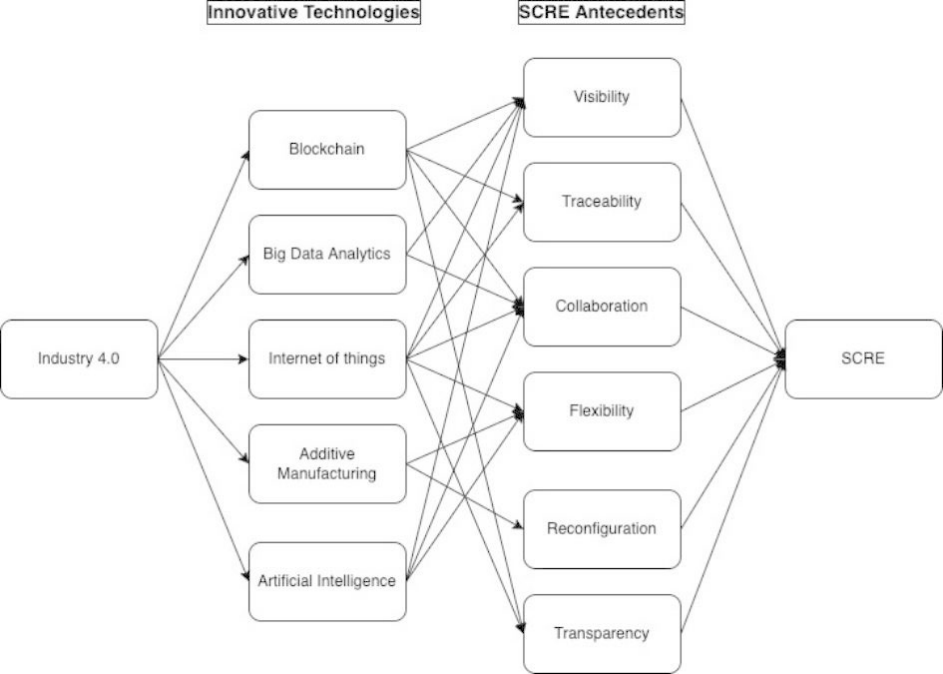
Industry 4.0 technologies and SCRE

The sample studies of this cluster also discuss the application of IoT in different sectors to make SCs more resilient. For example, food supply chains are an integral part of every economy therefore to avoid supply disruptions, evaluation of risks beforehand and assure continuity of operations, Ali et al., (2019) suggested adopting IoT enabled technologies to monitor product history and origin tracking. Hence, this helps in improving the visibility and risk transparency in the SCs (Birkel & Hartmann, 2020). Whereas, adopting IoT in the retail SCs offer more benefits in terms of creating flexibility, visibility, adaptation, and reduced operational risks (Gao et al., 2020). Figure 2 shows the connection of IoT with different SCRE enablers. Similarly, IoT-enabled technology might be used in other industrial sectors, for instance, pharmaceuticals and construction, to develop internal capacities to predict and mitigate supply chain interruptions (Rejeb et al., 2020).

In a nutshell, the current research depicts that IoT-enabled technological innovation supports data analytics and thereby capability to early detect disruptive behaviours which results in sooner preventive measures to assure business continuity and resiliency. Further, innovative technologies also protect supply chains against market dynamism and uncertainties. However, the application of innovative technologies is not without challenges. That is, IoT is an integrated system, and therefore to provide the privacy and security of supply networks during information exchange business enterprises also need to develop cybersecurity measures.

### Cluster 2: Blockchain, Industry 4.0, big data analytics, and resilience

Cluster 2 represents red and green clusters that are more interconnected and constitute closely associated keywords. As such, we treated these clusters together while labelling the key themes. The most frequently co-occurring keywords in these two clusters include industry 4.0, big data analytics, data mining, blockchain, resilience, forecasting, decision-making process, among others. Looking at the studies behind these keywords, we classified red and green clusters as ‘blockchain, Industry 4.0, and SCRE’. The three major streams of literature that have emerged in these clusters include, blockchain, Industry 4.0, and big data analytics and their relationships with SCRE.

The first stream of research examines blockchain-enabled data sharing and the resulting SCRE. For instance, Lohmer et al., (2020) stressed the use of blockchain technology strengthens the resilience of the supply chains and substantially reduces disruption propagation by developing a transparent, visible, and automated data exchange mechanism. Likewise, Babich & Hilary (2020), Mylrea & Gourisetti (2018), and Queiroz et al., (2020) added that blockchain technology enhances visibility, validation, transparency, secure transactions, traceability, and cyber-security which then improves the resiliency in the network.

Prior research also highlighted that blockchain technology provides the immutability, auditability, and provenance that secures and traces data hence providing visibility beyond the tier 1 supply networks (Kamble et al., 2020; Shahzad et al., 2020; Lin & Zhang, 2020). Further, within the supply network, trading partners extensively interact with each other and intersect business functions across the national boundaries; thus, prone to vulnerabilities. Accordingly, Min (2019) argued that since blockchain technology is a digitally distributed ledger and possesses a network verification process, it is capable to enhance SCRE in times of uncertainty through data sharing, asset tracking, automating order fulfilment, and cybersecurity. That is, blockchain possesses the capability to transform the traditional risk management process, mainly focused on mitigation of risk, to blockchain-enabled resilience management by having a proactive nature, uncovering invisible and hidden risks, and providing multi-layered protection. Besides the operational disruptions, the literature has also discussed the significance of blockchain during the current pandemic (Lohmer et al., 2020; Li et al., 2021; Bhaskar et al., 2020). During the COVID-19 induced lockdown, many businesses observed disruptions such as sudden spikes in demand causing shortages in life-saving drugs, personal and protective equipment, ventilators, etc. (Ali et al., 2022). This has exposed how fragile the current supply network systems requiring to reimagining the structural design. In this vein, Bhaskar et al., (2020) suggested that lean intensive approaches alone would not be useful during the pandemic. Therefore, to mitigate the ripple effects during high impactful disruptive events and ensure a continuous supply, they proposed an integrated framework along with blockchain technology to improve governance, reduce inefficiencies and smoothen supply chain processes among the public healthcare institutions. Transparency attained through blockchain technology would play a significant role in rebuilding the disrupted supply networks, as it would allow trading partners, regulators, and consumers to verify information (Hewett et al., 2020). Through a synchronized process, blockchain technology ensures the transparency of reliable and secure data using a set of predetermined criteria. Further, the application of blockchain technology to mitigate the disruptions and improve resiliency is also studied in different industry settings, for example, agriculture (Kamble et al., 2020; Bodkhe et al., 2020); food and beverage (Ali et al., 2021; Emmanouilidis and Bakalis, 2020); and healthcare (Jayaraman et al., 2018; Bell et al., 2018).

The second stream of research in these two clusters focuses on Industry 4.0 enabled data analytics and SCRE. From this perspective, Katsaliaki & Mustafee (2019) discussed the impact of Industry 4.0 supported data exchange in creating resiliency against ripple effects. They identified that Industry 4.0 with customized configuration creates higher market flexibility and shorter lead times at the cost of mass production, thereby resulting in higher responsiveness. Likewise, Fragapane et al., (2020) note that Industry 4.0 enables data exchange to create adaptive systems, which is a significant ability to maintain the supply chain amidst disruption. Ralston & Blackhurst (2020) espoused that Industry 4.0 enhances the firm’s adaptation and reconfiguration capabilities, which contributes to enhanced resilience by improving performance throughout the whole SC and assisting organisations in dealing with unanticipated circumstances. Besides, the beneficial effects of Industry 4.0, data analytics and SCRE, researchers (e.g., Kaur & Singh 2020; Sharma et al., 2020) have also studied the resilient supplier evaluation and selection methodology in Industry 4.0 environment to identify technologically capable suppliers under different disaster scenarios. Additionally, Ramirez-Pena et al. (2020) argued that Industry 4.0 technologies coupled with lean and resilient paradigms create visibility and connectivity for shipbuilding SCs. Likewise, Ivanov (2017) and Dolgui et al., (2018) explored that an extensive SC structure possesses high complexities and uncertainties, and as such, adoption of Industry 4.0 along with cyber-physical systems will facilitate predicting the performance of manufacturing plants and supply chains. Thus, it will help in devising recovery strategies for the negative event in advance. Industry 4.0 supported data integration between supply chain partners, helps in improving the material delivery reliability and reduces the information distortions (Ali et al., 2021), hence improving the supply chain continuity. For example, information interchange via Industry 4.0 enabled data analytics allows enterprises to greatly enhance demand prediction accuracy, lowering the impact of any supply-demand discrepancy and therefore preventing supply chain operational interruptions. Its application is adopted in multiple industries to reduce disruptions and improve supply chain resilience, such as agriculture, automobile, pharmaceuticals, shipbuilding Kumar et al., 2010; Ralston & Blackhurst, 2020; Kaur & Singh, 2020; Sharma et al., 2020; Jirsak, 2018; Ramirez-Pena et al., 2020; Chandriah and Raghavendra, 2019).

The third stream of research offers insights on big data analytics (BDA) and SCRE. BDA refers to an integration of structured and non-structured data from multiple sources in the supply network (suppliers, manufacturing plants, warehouses, customers, etc.) to reach processable information for efficient decision making (Raut et al., 2021). From the SCRE perspective, BDA is discussed in anticipating disruption occurrences, reaction planning, and real-time controlling (Dubey et al., 2019; Singh & Singh, 2019; Mandal, 2019), for example, explored the extent to which a firm’s existing technological infrastructure capabilities and big data analytics add value in responding to disruptive events. Similarly, Dubey et al., (2019) studied the big data analytics capability to enhance information processing capacity, control ripple effects, and thereby improve resilience. Whereas other empirical studies have highlighted the role of big data analytics on the LARG (lean, agile, resilient, and green) paradigm (Raut et al., 2021); different big data analytics related capabilities on multiple resilient dimensions (Mandal, 2019); and big data analytics capability on multiple hospital supply chain integration dimensions to respond COVID-19 related crises (Yu et al., 2021). Still, others explored how big data could be utilized in disaster-prone areas to assure resiliency (Papadopoulos et al., 2017; Lee et al., 2016).

In summary, the structural design of supply networks has massively expanded over time, thereby increasing the uncertainty and probability of disruption propagation. In this scenario, the firm’s decision-making ability if powered by innovative technologies (Industry 4.0, blockchain, and big data analytics) may pave the way for managing disruptions risks and controlling the ripple effects in the network. These innovative technologies with their fast-paced ability enable firms to adjust their manufacturing or service structure by promptly responding to new changes in the environment. Since these technologies have real-time visibility capability, therefore, firms are inclined towards adopting them due to the adaptability and reconfiguration capability to deal with unexpected events. Figure 2 also shows the connection of Industry 4.0 enabled BDA and blockchain technologies with the antecedents of SCRE.

### Cluster 3: 3-D printing, artificial intelligence, and SCRE

The most frequently co-occurring keywords in the yellow cluster include 3D-printing, artificial intelligence, COVID-19, and supply chain disruption, among others. Referring to the literature related to these terms, we classified yellow clusters into two main themes: (1) 3D-printing (additive manufacturing) and SCRE, and (2) artificial intelligence and SCRE.

The first stream of research sheds light on the nexus of additive manufacturing (AM) and SCRE. A few of the key approaches of AM discussed in the extant literature are rapid prototyping, rapid manufacturing, rapid tooling, and mass customization, among others (Van der Elst et al., 2020; Verboeket & Krikke 2019). Researchers highlighted that adopting additive manufacturing (AM) enables firms to bring the production near to the demand hence making it more responsive and adaptable to sudden market changes than traditional manufacturing systems (Ivanov et al., 2019; Verboeket & Krikke, 2019). Particularly, the authors have stressed the use of 3D printing by the manufacturing companies in responding COVID-19 pandemic crisis to assure uninterrupted medical supplies (Longhitano et al., 2021; Salmi et al., 2020). This study looked at how AM ensured continuous supply by producing locally and on-demand to deal with the disruptions created by COVID-19, such as shortages in medical supply and services. Several resilient practices have been recognized using AM (Naghshineh & Carvalho, 2020), for instance: the role of flexibility, robustness, and resilience in adopting AM technology (Chiu & Lin, 2016); how AM improves SC readiness and responsiveness in a disaster-prone area (Meisel et al., 2016); and AM’s role in reducing structural SC complexity (Ivanov, 2018). These authors analyzed AM characteristics and find out that it leads to increased flexibility, shorter lead times, financial strength, higher responsiveness, and efficient control of maintenance, repair, and operating supplies (MROs)

The second stream of manuscripts is on the relationship between artificial intelligence (AI) and SCRE. Some of the approaches which are used in AI are genetic algorithms, swarm optimization, artificial bee colony, agent-based systems, fuzzy logic and programming, among others (Kumar et al., 2010; Frayret, 2011; Baryannis et al., 2019; Pramanik et al., 2020). In this vein, Baryannis et al., (2019) and Rajesh (2020) examined the application of AI in quantifying the resilient strategies for identifying, assessing, mitigating and monitoring the disruptive events in the supply networks. To increase the resiliency in agri-food supply chains, Zavala-Alcívar et al., (2020) analyzed the application of AI techniques in the supplier selection and evaluation process. Whereas Kumar et al., (2010) adopted AI-related approaches to minimize supply chain costs associated with different types of disruptions. Further, to anticipate the disruptive events Baryannis et al., (2019) proposed an AI-based framework that will be useful in the decision making to shield against disruptions. Similarly, Reyes et al., (2020) adopted AI-related computational techniques to investigate their applicability in identifying supply network disruptions. AI approaches have also been used to predict cargo theft and secure the infrastructure, particularly in the railways’ transport case (Lorenc et al., 2020). Researchers have also evaluated inventory and sourcing strategies to manage supply uncertainties through the AI approaches (Pramanik et al., 2020); and explored the potential of AI techniques for supplier selection to assess their responsiveness against disruptive events (Zhao & Yu, 2011). Lastly, Rodríguez-Espíndola et al., (2020) mentioned that AI-driven computational techniques provide reliable and updated forecasts in real-time, thus creating end-to-end visibility and coping uncertainty.

Overall, the literature on additive manufacturing and artificial intelligence for SCRE is emerging and nascent. In the first stream, there is a surge of articles on the use of additive manufacturing for high impactful disruptions like the COVID-19 pandemic. However, the second stream focuses on the identification of disruptive impacts and suggestions for business continuity. It is also expected that these sophisticated technologies will help in reducing the structural complexity and uncertainties in SCs, by using fewer components and materials in the product design stage. Also, these technologies would reduce the impact of geopolitical risks by producing materials locally.

In a nutshell, the review of extant literature shows the connections between Industry 4.0 technologies and key antecedents of SCRE (see Fig. 2). Building upon the literature, it can be expected that the adoption of various technologies (blockchain, big data analytics, IoT, additive manufacturing and AI) would support supply chain resilience through improvement in visibility, traceability, collaboration, flexibility, reconfiguration, and transparency.

### Evolution of research on the topic

**Fig. 3:**
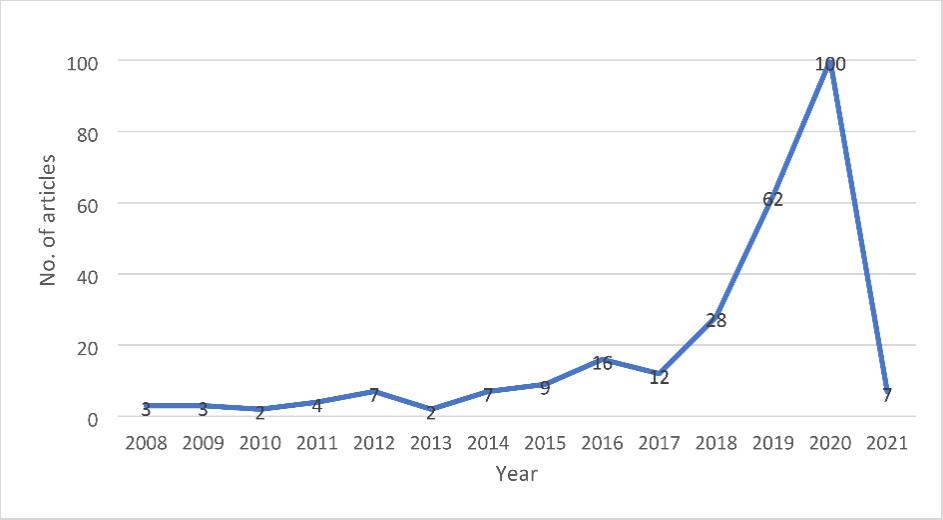
Evolution of research on innovative technologies and SCRE

This section presents the distribution of studies from 2008 to 2021 to track the progression of research on this under-studied topic. Figure 3 shows that the topic of innovative technology, data analytics and SCRE has been evolving since 2008, albeit with an inconsistent pattern of growth. From 2008 to 2013, development in this subject area was somewhat sluggish and erratic.

We can see an uneven trend of growth from 2014 to 2017. Whereas, from 2017 and onwards, there is a significant growth in the number of studies which have exponentially grown till the 2020-year end. The highest number of studies (100) were observed in 2020. This demonstrates the topic’s high recognition and popularity among the operations and supply chain management research community. Because the search for articles was conducted on 04 January 2021, we only found 7 items with a sharp decline in the trend line.

### Knowledge trajectories

**Fig. 4:**
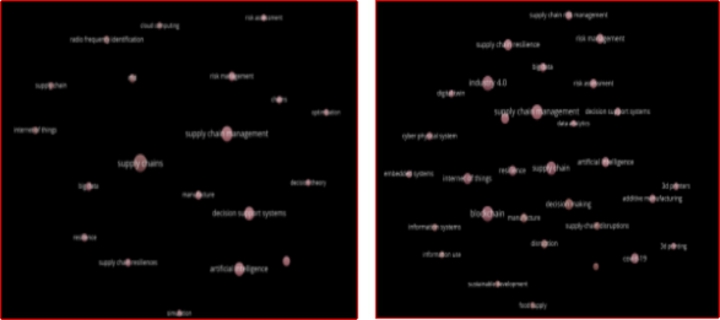
Knowledge trajectories between two main periods (2008–2017 and 2018–2021)

To observe the trajectories of knowledge over the past two, keywords clusters were created for two different periods i.e., 2008–2017 and 2018–2021 (see Fig. 4). The unequal distribution of years between the two periods is attributed to the uneven development of the research topic. That is, while the literature on the intersection of innovative technologies and SCRE began to develop in 2008, a visible change was noticed from 2018.

We have noticed a significant shift in the knowledge flow between the two time periods shown in Fig. 4. From 2008 to 2017, popular keywords were radio frequency identification, RFID, internet of things, big data, cloud computing, resilience, supply chains, risk management, risk assessment, artificial intelligence, and decision support systems.

Whereas, Industry 4.0, blockchains, additive manufacturing, 3-D printing, cyber-physical system, digital twin, and covid-19 are among the new themes, from 2018 to 2021. The remaining keywords, on the other hand, are comparable, but their node size, which represents several studies, is significantly bigger than in the previous period (2008–2017). Internet of things, big data analytics, blockchains, risk management, and SCRE are a few examples. The additional keywords and larger node sizes represent the emergence of innovative technologies and SCRE. This information could assist the early career researchers and prospective researchers to understand the dynamics of recent research while choosing a new research project.

### Classification of research methods

**Fig. 5:**
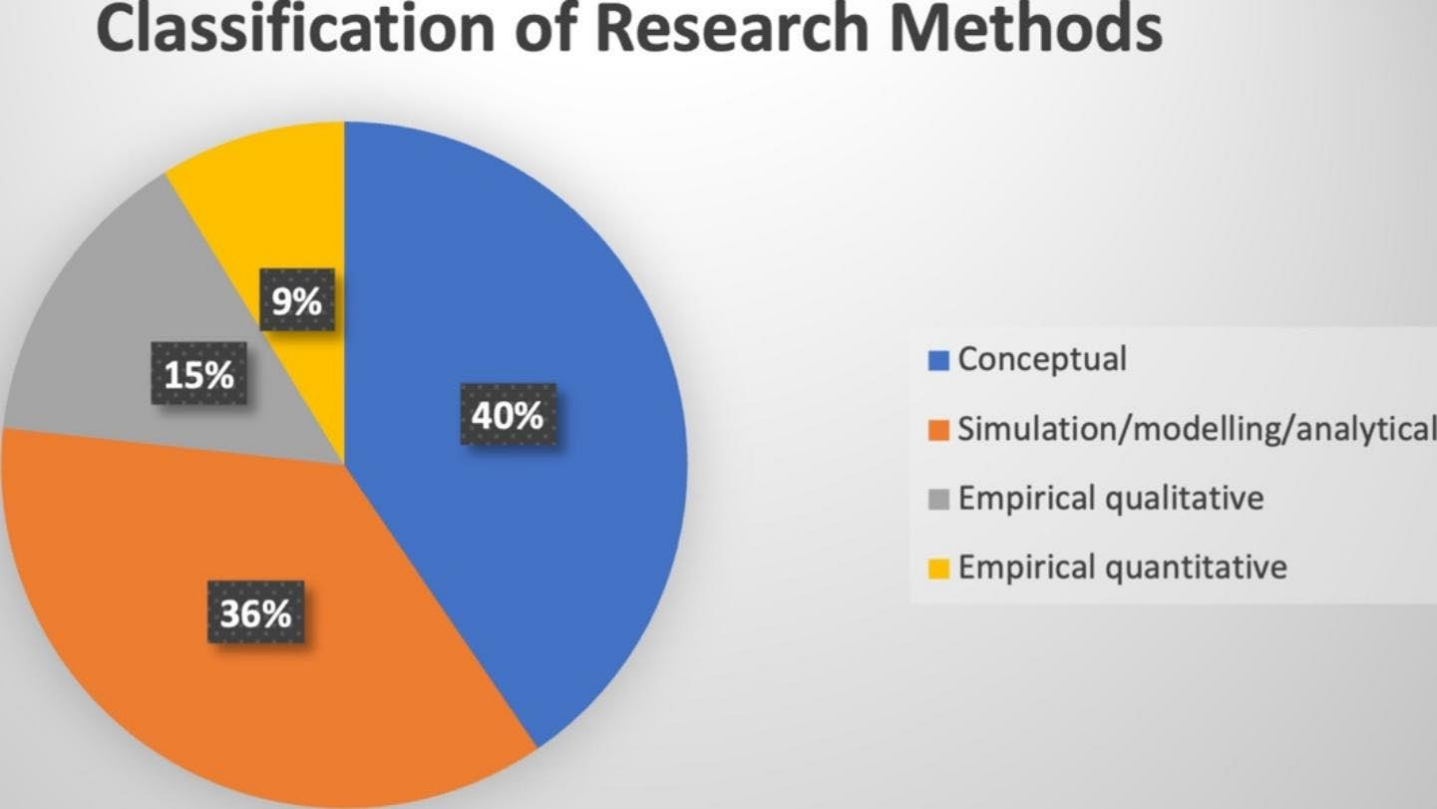
Classification of research methods

The sampled studies (262) in our review present different methodological perspectives (see Fig. 5). These include: 105 (40%) studies consist of *conceptual/literature reviews* (e.g., Ivanov & Dolgui 2019; Min, 2019; Verboeket & Krikke, 2019; Hosseini & Ivanov, 2020); 94 (*36%) studies are simulation/modelling/analytical* (e.g., Kumar et al., 2010; Ivanov et al., 2013; Bottani et al., 2019; Lohmer et al., 2020): 39 (*15%) articles comprise of empirical qualitative* (e.g., Kask & Öberg 2019; Ralston & Blackhurst 2020); *and 9% (24) studies constitute empirical quantitative* (e.g., Papadopoulos et al., 2017; Wamba & Queiroz, 2020; Dubey et al., 2020).

Clearly, a major portion of the literature (76%) is based on conceptual frameworks plus simulation and analytical papers, most of which lack real-world data. Whereas only 24% of the studies adopted empirical methodologies, both qualitative and quantitative. There are fewer studies in quantitative empirical papers (survey-based research) than in qualitative empirical papers, indicating a substantial difference.

## Discussion and future research avenues

The review of the literature shows a significant increase in the literature on innovative technologies, data analytics and SCRE. The descriptive evaluation of 262 eligible articles in this systematic literature review highlighted that the growth rate of research publications on innovative technologies, data analytics, and supply chain disruption and resilience has exponentially grown. We found that IoT facilitates the design of intelligent and robust SCs by enabling real-time information exchange, and improved cooperation among business partners in the network. Also, the data acquired through IoT technology could be used in forecasting abrupt spikes in demand, preparing and responding to the sudden changes timely, hence protecting supply chains from extremely disruptive shocks. Furthermore, rising worldwide demand has prompted businesses to diversify their product offerings and expand into new markets. Hence, leads towards extended supply chains, which cause unpredictability and dynamism in the external environment (Azadegan et al., 2013). In this situation, the firm’s decision-making abilities, influenced by innovative technologies (Industry 4.0, blockchain, and big data analytics), may pave the way for managing and controlling disruption risks and ripple effects in the network. Our bibliometric review also highlighted that using AM allows businesses to shift production closer to demand, making them more responsive and flexible to market changes than traditional manufacturing processes. It was also noted that businesses during the COVID-19 pandemic adopted 3D printing, producing face masks and shields, to ensure meet consumer demand. This review further highlights the AI-related approaches, i.e., agent-based systems, swarm optimization, artificial bee colony, etc., used by the researchers to understand the complex behaviour of supply network structures towards resilience and robustness (Giannoccaro & Iftikhar, 2020).

Based on this study we explored the significance of SCRE antecedents with the innovative technologies enabled data analytics. We found that the usage of innovative technologies by researchers has evolved within the supply chain disruption and resilience domain from RFID to blockchain and cyber-physical systems. Following the development in this area, different industries in manufacturing and service have been investigated separately to develop resilience measures (Belhadi et al., 2021; Mubarik et al., 2021). Nonetheless, we recognized that there is a substantial gap in performing empirical research on issues surrounding the nexus of innovative technologies and supply chain disruptions and resilience. During the review of the literature, plenty of opportunities for future research emerged on the confluence of innovative technologies, data analytics and SCRE. We have highlighted some of them with specific themes in the below section. Table 2 summarizes the research themes along with the future research questions.

### Complexity, innovative technologies, and resilience

The extant literature identified different forms of SC complexities that could have both detrimental and beneficial effects on a firm’s business value and resilience (Bode & Wagner, 2015; Chowdhury et al., 2019). However, the following important questions remain unanswered: (1) how do different innovative technologies (big data analytics, blockchain, IoT, artificial intelligence, etc.) interact with multiple forms of complexities (static, dynamic, upstream, internal, and downstream) to leverage SCRE? (2) How does data analytics interact with different dimensions of SC complexities, and how simultaneously it can improve traceability and transparency in the supply network?

While blockchain has received a great deal of attention in the literature (Wang et al., 2020; Kurpjuweit et al., 2019; Min 2019; Lohmer et al., 2020), little is known about the role of network orchestrators that blockchain-enabled data exchange could display in highly complex supply chains.

### Blockchain-enabled data analytics and SCRE

In contemporary SCs, procurement frauds are amid the significant menaces and vulnerabilities that disrupt the financial flows among the network partners. However, blockchain-based data analytics and transactions assure transparency and fraud reduction (Wang et al., 2019; Guerar et al., 2020). IBM blockchain platform, for example, developed distributed digital ledger in which transaction data is shared among the network and constantly reconciled hence it’s hard to alter the information by the stakeholders. Barclay – the second-largest bank in the UK - has been successfully using blockchain in its banking system. Other examples include Unilever and Walmart, which are using IBM blockchain in procurement thus reducing risks of, tampering, counterfeiting and fraud. We suggest researching this new phenomenon to bring vital insights into how blockchain technologies facilitate financial communication across the network through analyses of data on business transactions, thereby reducing frauds and counterfeiting.

The dynamics of understudied innovative technologies are different, as revealed during the literature review (Ivanov et al., 2019; Ivanov and Dolgui, 2020; Baryannis et al., 2019). However, we do not know the synergistic effects of these innovative technologies (e.g., blockchain in AI environment, big data analytics, and cyber-physical systems) on data analytics capabilities and SCRE. This topic could be studied by considering different contextual variables, such as competition intensity, technological complexity, or bargaining power. Further, what other resources, tangible, or intangible, would be required by the firms to achieve the beneficial effect of resiliency, an area that warrants further research.

The analysis of the literature depicts that past research predominantly focuses on blockchain’s adoption challenges in the traditional manufacturing industries (Wamba & Queiroz, 2020; Queiroz & Wamba, 2019; Moktadir et al., 2019), leaving the gap for research on other important contexts. Particularly, while the importance of food supply chains has been increasingly recognised during the COVID-19 outbreak, there is a lack of broad empirical evidence if and how blockchain technology can be effectively used to deal with the pandemic and ensure a smooth supply of food. As such, we suggest large-scale research on the application of blockchain in the agri-food supply chains specifically on SMEs in emerging and developing economies. The adoption of these innovative technologies would secure the financial flow of SMEs, leverage their productivity, and assure business continuity, transparency, and visibility through ‘disintermediation’ (Wang et al., 2019).

### SC mapping through innovative technologies enabled data analytics

SC mapping is considered one of the significant sources to assure supply resiliency and business continuity in complex supply networks (Gardner & Cooper, 2003). It provides the firm with a holistic visualization of its entire supply network (Swift et al., 2019). Owing to the significant disruptions caused by the global pandemic (COVID-19), Choi et al., (2020), and Mubarik et al., (2021) stress SC mapping, which could enable to a better understanding of the network structure in which a firm operates (Giannoccaro & Iftikhar, 2020). Given this, a fruitful future research avenue is to investigate the role of innovative technologies enabled data analytics in SC mapping to enable resilience.

### Innovative technologies, data analytics and cyber-resilience

A compelling body of literature identifies the vulnerabilities among the traditional SC flows (Ali and Golgeci, 2019; Ivanov 2017; Xu et al., 2020). Similarly, digital SCs are not risk-free and possess significant threats, impacting different industrial sectors (Büyüközkan & Göçer, 2018). To achieve cyber resilience, firms need to adopt a holistic approach (Boyes, 2015). Therefore, it is of utmost importance for future researchers to explore how the dark sides of innovative technologies could be minimized, what enabling factors to assure cyber resiliency firms need to develop, how digital platforms will be kept secured particularly in the critical infrastructures and maritime sectors in the wake of disruptions. This will further build confidence among the customers and suppliers to adopt technology driven SC solutions. Future researchers could also identify the critical managerial skills required to make data analytics-based decisions and manage the cyber resilience operations, grounded in different theoretical frameworks, such as technology acceptance model (TAM), technology organization environment (TOE) framework, or unified theory of acceptance and use of technology (UTAUT) (Jeyaraj & Dwivedi, 2020; Tao et al., 2020).

### Innovative technologies, relational governance, and resilience

The presence of trust is considered a significant organizational capability to leverage resilience in a disruptive environment (Ali and Golgeci, 2019). However, it also has its limitations, such as it is expensive, time-consuming, and difficult to manage under high structural complexity (Capaldo & Giannoccaro, 2015; Galvin et al., 2021). While adopting innovative technologies and deciding based on data analytics, firms are not obliged to trust their partners as they used to, it is integrated into these blockchain platforms (Wang et al., 2019). Therefore, an interesting avenue for future research could be to investigate the degree to which reliance on partners via trust mechanisms would be effective in disruptive environments under the wave of innovative technologies to improve resilience in the network. It would be insightful to explore whether adopting innovative technologies would lessen the need for developing interorganizational relationships or protect SC partners against adopting any opportunistic behavior. The extant literature suggests that relational capabilities reduce uncertainty and risk and enhance resilience, however, the extent to which relational attributes/capabilities be effective under digital transformation while mitigating SC disruptions, is an area not well understood.

### Innovative technologies and reshoring

Owing to the severe disruptions caused by the COVID-19, a significant discussion emerged around bringing manufacturing closer to the demand and making SCs shorter (Choi et al., 2020). As such, future researchers could empirically explore how innovative technologies supported by data analytics would affect the firm’s location decisions or/and how they will enable viable reshoring to further heighten the responsiveness to market dynamics and uncertainty. Further, the impact of different industrial sectors could be explored, such as the textile industry, the high-tech sector, consumer goods, electronics and electrical, and other manufacturing industries.

### Innovative Technologies and SC disruption management

Our review of literature depicts those different types of disruption risks that have been comprehensively categorized (Craighead et al., 2007; Giannoccaro & Iftikhar, 2020) along with different SC disruption management phases and strategies (Macdonald & Corsi, 2013). In doing so, most of the prior literature explored how SC disruptions management strategies reduce the vulnerabilities and mitigate the disruptive impact.

However, the role of different innovative technologies in different SC disruption management phases, disruption detection, reaction, recovery, and learning to improve post-disruption supply chain recovery, is an area that warrants further investigation.

**Table 2:** Research themes and future research questions

Research theme	Future Research Questions
Supply chain complexities, innovative technologies & resilience.	Q1. How do different innovative technologies (big data analytics, blockchain, IoT, artificial intelligence, etc.) interact with multiple forms of complexities (static, dynamic, upstream, internal, and downstream) to leverage SCRE?
	Q2. How does data analytics interact with different dimensions of SC complexities, and how simultaneously it will impact the traceability in the supply network?
	Q3. What is the role of blockchain-enabled data exchange in network orchestration for highly complex supply chains (i.e., automotive sector, fashion industry, etc.), and at the same time how it would improve transparency beyond 1st tier suppliers?
Blockchain-enabled data analytics and SCRE	Q1. What is the role of blockchain-based data analytics to protect financial flows among the network partners to assure resiliency in global value chains?
	Q2. What are the synergistic effects of innovative technologies on data analytics capabilities and SCRE?
	Q3. How blockchain technology can be effectively used to deal with the highly uncertain environment and ensure resiliency in agri-food supply chains in certain contexts (i.e., SMEs, emerging & developing economies).
Supply chain mapping and innovative technologies	Q1. What is the role of innovative technologies enabled data analytics in SC mapping to enable resilience?
Innovative technologies, data analytics and cyber resiliency	Q1. What enabling factors to assure cyber resiliency firms need to develop, how digital platforms will be kept secured particularly in the critical infrastructures and maritime sectors?
	Q2. What critical managerial skills are required to make data analytics-based decisions and manage the cyber resilience operations, grounded in different theoretical frameworks?
Innovative technologies and relational governance	Q1. How effective it would be for firms in supply chains while adopting innovative technologies to operate in a trustless marketplace under a disruptive environment?
Innovative technologies and reshoring	Q1. How innovative technologies enabled data analytics would affect the firm’s location decisions or/and how they will enable viable reshoring to further heighten the responsiveness to market dynamics and uncertainty?
Innovative technologies and SC disruption management	Q1. What is the role of different innovative technologies at different SC disruption management phases to improve post-disruption supply chain recovery?

## Conclusions

Unprecedented disruptive incidents have increasingly attracted academics’ interest in innovative technologies, data analytics and SCRE. This study aimed to review the existing literature at the nexus of innovative technologies, data analytics and SCRE. We found that innovative technologies (big data analytics, IoT, Industry 4.0, additive manufacturing, blockchain, artificial intelligence, etc.) supported data analytics can play a significant role in anticipating disruptive events, reducing their impacts and assuring business continuity. Despite considerable contributions, many past studies are still conceptual and lack empirical corroboration. In particular, there is a severe shortage of mixed methods research, which is considered important to support the rigor and clarity of the underlying relationships. We have recognized several promising opportunities for more impactful future research on this interesting yet underexplored topic of research.

Aside from the interesting findings, this study has several limitations, just like any other literature review. Despite using a broad set of search keywords on innovative technologies and SCRE to collect as many publications as possible, it’s possible that a few of the articles were overlooked unintentionally. However, our considerably large sample size (262 articles) assures that the findings are a fair reflection of the research conducted around the topic. We identified four research clusters by employing bibliometric analysis; however, future researchers might use other methods, such as homogeneity analysis using alternating least squares (HOMALS) to cross-check our findings. Future researchers may also improve the content analysis by adopting the multiple correspondence analysis (MCA) approach. Finally, we provide significant insights on the SCRE research and advance knowledge from the standpoint of digital innovation and data analytics; our research agenda has the potential to spark further discussion around this topic.

## References

[CR1] Akter S, Motamarri S, Hani U, Shams R, Fernando M, Babu MM, Shen KN (2020). a. Building dynamic service analytics capabilities for the digital marketplace. Journal of Business Research.

[CR2] Akter S, Gunasekaran A, Wamba SF, Babu MM, Hani U (2020). b. Reshaping competitive advantages with analytics capabilities in service systems. Technological Forecasting and Social Change.

[CR3] Al-Talib M, Melhem WY, Anosike AI, Reyes JAG, Nadeem SP (2020). Achieving resilience in the supply chain by applying IoT technology. Procedia CIRP.

[CR4] Ali I, Arslan A, Chowdhury M, Khan Z, Tarba SY (2022). Reimagining global food value chains through effective resilience to COVID-19 shocks and similar future events: A dynamic capability perspective. Journal of Business Research.

[CR5] Ali, I., Arslan, A., Khan, Z., & Tarba, S. Y. (2021). The Role of Industry 4.0 Technologies in Mitigating Supply Chain Disruption: Empirical Evidence From the Australian Food Processing Industry. *IEEE Transactions on Engineering Management*, 1–11. 10.1109/TEM.2021.3088518

[CR6] Ali A, Mahfouz A, Arisha A (2017). "Analysing supply chain resilience: integrating the constructs in a concept mapping framework via a systematic literature review". Supply Chain Management.

[CR7] Ali I, Gölgeci I (2019). Where is supply chain resilience research heading? A systematic and co-occurrence analysis. International Journal of Physical Distribution & Logistics Management.

[CR8] Ali I, Gölgeci I (2021). Managing climate risks through social capital in agrifood supply chains. Supply Chain Management: An International Journal.

[CR9] Ali, I., Golgeci, I., & Arslan, A. (2021). Achieving resilience through knowledge management practices and risk management culture in agri-food supply chains. Supply Chain Management: An International Journal, ahead-of-print(ahead-of-print). 10.1108/SCM-02-2021-0059

[CR10] Ali, I., & Govindan, K. (2021). *Extenuating operational risks through digital transformation of agri-food supply chains* (pp. 1–13). Production Planning & Control

[CR11] Ali SM, Moktadir MA, Kabir G, Chakma J, Rumi MJU, Islam MT (2019). Framework for evaluating risks in food supply chain: Implications in food wastage reduction. Journal of cleaner production.

[CR12] Ali I, Aboelmaged MGS (2022). Implementation of supply chain 4.0 in the food and beverage industry: perceived drivers and barriers. International Journal of Productivity and Performance Management.

[CR13] Ali I, Sultan P, Aboelmaged M (2021). A bibliometric analysis of academic misconduct research in higher education: Current status and future research opportunities. Accountability in Research.

[CR14] Arslan, A., Cooper, C., Khan, Z., Golgeci, I., & Ali, I. (2021). Artificial intelligence and human workers interaction at team level: a conceptual assessment of the challenges and potential HRM strategies. International Journal of Manpower, ahead-of-print(ahead-of-print). 10.1108/IJM-01-2021-0052

[CR15] Ataseven C, Nair A (2017). Assessment of supply chain integration and performance relationships: A meta-analytic investigation of the literature. International journal of production economics.

[CR16] Ateş, M. A., Suurmond, R., Luzzini, D., & Krause, D. (2021). Order from Chaos: A Meta-analysis of Supply Chain Complexity and Firm Performance.Journal of Supply Chain Management, e12264

[CR17] Azadegan A, Patel PC, Zangoueinezhad A, Linderman K (2013). The effect of environmental complexity and environmental dynamism on lean practices. Journal of operations management.

[CR18] Babich V, Hilary G (2020). OM Forum—Distributed ledgers and operations: What operations management researchers should know about blockchain technology. Manufacturing & Service Operations Management.

[CR19] Baryannis G, Validi S, Dani S, Antoniou G (2019). Supply chain risk management and artificial intelligence: state of the art and future research directions. International Journal of Production Research.

[CR20] Belhadi A, Kamble S, Jabbour CJC, Gunasekaran A, Ndubisi NO, Venkatesh M (2021). Manufacturing and service supply chain resilience to the COVID-19 outbreak: Lessons learned from the automobile and airline industries. Technological Forecasting and Social Change.

[CR21] Belhadi, A., Mani, V., Kamble, S. S., Khan, S. A. R., & Verma, S. (2021). *Artificial intelligence-driven innovation for enhancing supply chain resilience and performance under the effect of supply chain dynamism: an empirical investigation* (pp. 1–26). Annals of Operations Research 10.1007/s10479-021-03956-x PMC7856338 33551534

[CR22] Bell, L., Buchanan, W. J., Cameron, J., & Lo, O. (2018). *Applications of blockchain within healthcare*. Blockchain in healthcare today

[CR23] Bemthuis, R. (2019, October). Business logic for resilient supply chain logistics. *2019 IEEE 23rd International Enterprise Distributed Object Computing Workshop (EDOCW)* (pp. 190–195). IEEE

[CR24] Burmester M, Munilla J, Ortiz A, Caballero-Gil P (2017). An RFID-based smart structure for the supply chain: Resilient scanning proofs and ownership transfer with positive secrecy capacity channels. Sensors.

[CR25] Bhaskar S, Tan J, Bogers ML, Minssen T, Badaruddin H, Israeli-Korn S, Chesbrough H (2020). At the Epicenter of COVID-19–the Tragic Failure of the Global Supply Chain for Medical Supplies. Frontiers in public health.

[CR26] Bodkhe, U., Tanwar, S., Bhattacharya, P., & Kumar, N. (2020). *Blockchain for precision irrigation: Opportunities and challenges*. Transactions on Emerging Telecommunications Technologies. e4059

[CR27] Birkel HS, Hartmann E (2020). "Internet of Things – the future of managing supply chain risks". Supply Chain Management.

[CR28] Bode C, Wagner SM (2015). Structural drivers of upstream supply chain complexity and the frequency of supply chain disruptions. Journal of Operations Management.

[CR29] Bottani E, Murino T, Schiavo M, Akkerman R (2019). Resilient food supply chain design: Modelling framework and metaheuristic solution approach. Computers & Industrial Engineering.

[CR30] Boyes H (2015). Cybersecurity and cyber-resilient supply chains. Technology Innovation Management Review.

[CR31] Büyüközkan G, Göçer F (2018). Digital supply chain: literature review and a proposed framework for future research. Computers in Industry.

[CR32] Capaldo A, Giannoccaro I (2015). How does trust affect performance in the supply chain? The moderating role of interdependence. International journal of production Economics.

[CR33] Chandriah, K. K., & Raghavendra, N. V. (2019, April). Architectural Framework for Industry 4.0 Compliance Supply Chain System for Automotive Industry. In Computer Science On-line Conference (pp. 107–116). Springer, Cham

[CR34] Chen L, Dui H, Zhang C (2020). A resilience measure for supply chain systems considering the interruption with the cyber-physical systems. Reliability Engineering & System Safety.

[CR35] Chiu MC, Lin YH (2016). "Simulation-based method considering design for additive manufacturing and supply chain: An empirical study of lamp industry". Industrial Management & Data Systems.

[CR36] Choi TY, Rogers D, Vakil B (2020). Coronavirus is a wake-up call for supply chain management. Harvard Business Review.

[CR37] Chowdhury MMH, Quaddus M, Agarwal R (2019). "Supply chain resilience for performance: role of relational practices and network complexities". Supply Chain Management.

[CR38] Colquitt JA, Zapata-Phelan CP (2007). Trends in theory building and theory testing: A five-decade study of the Academy of Management Journal. Academy of management journal.

[CR39] Craighead CW, Blackhurst J, Rungtusanatham MJ, Handfield RB (2007). The severity of supply chain disruptions: design characteristics and mitigation capabilities. Decision sciences.

[CR40] Cui P, Dixon J, Guin U, Dimase D (2019). A blockchain-based framework for supply chain provenance. IEEE Access.

[CR41] Cui, Y. (2015). Improving supply chain resilience with employment of IOT. In International Conference on Multidisciplinary Social Networks Research (pp. 404–414). Springer, Berlin, Heidelberg

[CR42] Ding L, Lam HK, Cheng TCE, Zhou H (2018). A review of short-term event studies in operations and supply chain management. International Journal of Production Economics.

[CR43] Dolgui A, Ivanov D, Sokolov B (2018). Ripple effect in the supply chain: an analysis and recent literature. International Journal of Production Research.

[CR44] Dubey R, Gunasekaran A, Bryde DJ, Dwivedi YK, Papadopoulos T (2020). Blockchain technology for enhancing swift-trust, collaboration and resilience within a humanitarian supply chain setting. International Journal of Production Research.

[CR45] Dubey, R., Gunasekaran, A., Childe, S. J., Wamba, F., Roubaud, S., D., & Foropon, C. (2019). Empirical investigation of data analytics capability and organizational flexibility as complements to supply chain resilience.International Journal of Production Research,1–19

[CR47] Emmanouilidis, C., & Bakalis, S. (2020, August). Digital Technology enablers for resilient and customer driven food value chains. In IFIP International Conference on Advances in Production Management Systems (pp. 649–657). Springer, Cham

[CR48] Fragapane, G., Ivanov, D., Peron, M., Sgarbossa, F., & Strandhagen, J. O. (2020). *Increasing flexibility and productivity in Industry 4.0 production networks with autonomous mobile robots and smart intralogistics* (pp. 1–19). Annals of operations research

[CR49] Frayret JM (2011). Multi-Agent System applications in the forest products industry. Journal of Science & Technology for Forest Products and Processes.

[CR50] Gao Q, Guo S, Liu X, Manogaran G, Chilamkurti N, Kadry S (2020). Simulation analysis of supply chain risk management system based on IoT information platform. Enterprise Information Systems.

[CR51] Gajek, S., Lees, M., & Jansen, C. (2020). *IIoT and cyber-resilience* (pp. 1–11). AI & SOCIETY

[CR52] Galvin P, Tywoniak S, Sutherland J (2021). Collaboration and opportunism in megaproject alliance contracts: The interplay between governance, trust and culture. International Journal of Project Management.

[CR53] Gardner JT, Cooper MC (2003). Strategic supply chain mapping approaches. Journal of business logistics.

[CR54] Gelenbe, E., Fröhlich, P., Nowak, M., Papadopoulos, S., Protogerou, A., Drosou, A., & Tzovaras, D. (2020). IoT Network Attack Detection and Mitigation. In 2020 9th Mediterranean Conference on Embedded Computing (MECO) (pp. 1–6). IEEE

[CR56] Giannoccaro, I., & Iftikhar, A. (2020). Mitigating ripple effect in supply networks: the effect of trust and topology on resilience.International Journal of Production Research,1–18

[CR57] Gölgeci I, Ali I, Ritala P, Arslan A (2022). A bibliometric review of service ecosystems research: current status and future directions. Journal of Business & Industrial Marketing.

[CR58] Guerar M, Merlo A, Migliardi M, Palmieri F, Verderame L (2020). A fraud-resilient blockchain-based solution for invoice financing. IEEE Transactions on Engineering Management.

[CR59] Hendry LC, Stevenson M, MacBryde J, Ball P, Sayed M, Liu L (2019). Local food supply chain resilience to constitutional change: the Brexit effect. International Journal of Operations & Production Management.

[CR60] Hewett, N., Søgaard, J. S., & Mølbjerg, R. W. (2020). This Is How Blockchain Can Be Used In Supply Chains To Shape A Post-COVID-19 Economic Recovery

[CR61] Hoberg K, Alicke K (2016). The customer experience. Supply Chain Management Review.

[CR62] Hohenstein NO, Feisel E, Hartmann E, Giunipero L (2015). Research on the phenomenon of supply chain resilience. International Journal of Physical Distribution & Logistics Management.

[CR63] Hosseini S, Ivanov D (2020). Bayesian networks for supply chain risk, resilience and ripple effect analysis: A literature review. Expert systems with applications.

[CR64] Iftikhar A, Purvis L, Giannoccaro I (2021). A meta-analytical review of antecedents and outcomes of firm resilience. Journal of Business Research.

[CR65] Iftikhar, A., Purvis, L., Giannoccaro, I., & Wang, Y. (2022). *The impact of supply chain complexities on supply chain resilience: the mediating effect of big data analytics* (pp. 1–21). Production Planning & Control

[CR67] Ivanov D (2017). Revealing Interfaces of Supply Chain Resilience and Sustainability: A Simulation Study. International Journal of Production Research.

[CR68] Ivanov D, Dolgui A (2019). Low-Certainty-Need (LCN) supply chains: a new perspective in managing disruption risks and resilience. International Journal of Production Research.

[CR69] Ivanov D, Dolgui A, Sokolov B (2013). Multi-disciplinary analysis of interfaces “supply chain event management–RFID–Control theory”. International Journal of Integrated Supply Management.

[CR70] Ivanov D, Dolgui A, Sokolov B (2019). The impact of digital technology and Industry 4.0 on the ripple effect and supply chain risk analytics. International Journal of Production Research.

[CR71] Jayaraman, R., AlHammadi, F., & Simsekler, M. C. E. (2018). Managing product recalls in healthcare supply chain. In 2018 IEEE International Conference on Industrial Engineering and Engineering Management (IEEM) (pp. 293–297). IEEE

[CR72] Jeyaraj A, Dwivedi YK (2020). Meta-analysis in information systems research: Review and recommendations. International Journal of Information Management.

[CR73] Ji J, Plakoyiannaki E, Dimitratos P, Chen S (2019). "The qualitative case research in international entrepreneurship: a state of the art and analysis". International Marketing Review.

[CR75] Kadadevaramth, R. S., Sharath, D., Ravishankar, B., & Mohan Kumar, P. (2020). A Review and development of research framework on Technological Adoption of Blockchain and IoT in Supply Chain Network Optimization. 2020 International Conference on Mainstreaming Block Chain Implementation (ICOMBI) (pp. 1–8). IEEE

[CR76] Kamalahmadi M, Mellat-Parast M (2016). Developing a resilient supply chain through supplier flexibility and reliability assessment. International Journal of Production Research.

[CR77] Kamble SS, Gunasekaran A, Sharma R (2020). Modeling the blockchain enabled traceability in agriculture supply chain. International Journal of Information Management.

[CR78] Kask J, Öberg C (2019). "Why “majors” surge in the post-disruptive recording industry". European Journal of Marketing.

[CR80] Kaur, H., & Singh, S. P. (2020). *Disaster resilient proactive and reactive procurement models for humanitarian supply chain* (pp. 1–14). Production Planning & Control

[CR81] Kozyrkov C (2020). To Recognize Risks Earlier, Invest in Analytics. HARVARD BUSINESS REVIEW.

[CR82] Kumar SK, Tiwari MK, Babiceanu RF (2010). Minimisation of supply chain cost with embedded risk using computational intelligence approaches. International Journal of Production Research.

[CR85] Lee, Y., Watanabe, K., & Li, W. S. (2016). Enhancing regional digital preparedness on natural hazards to safeguard business resilience in the Asia-Pacific. In International Conference on Information Technology in Disaster Risk Reduction (pp. 170–182). Springer, Cham

[CR86] Li CZ, Chen Z, Xue F, Kong XT, Xiao B, Lai X, Zhao Y (2021). A blockchain-and IoT-based smart product-service system for the sustainability of prefabricated housing construction. Journal of Cleaner Production.

[CR87] Lin, C., & Zhang, Z. W. (2020). A Two-Tier Blockchain Architecture for the Digital Transformation of Multilateralism. In 2020 IEEE 91st Vehicular Technology Conference (VTC2020-Spring) (pp. 1–5). IEEE

[CR88] Lohmer J, Bugert N, Lasch R (2020). Analysis of resilience strategies and ripple effect in blockchain-coordinated supply chains: An agent-based simulation study. International journal of production economics.

[CR90] Lorenc A, Kuźnar M, Lerher T, Szkoda M (2020). Predicting the Probability of Cargo Theft for Individual Cases in Railway Transport. Tehnički vjesnik.

[CR91] Mandal S (2019). "The influence of big data analytics management capabilities on supply chain preparedness, alertness and agility: An empirical investigation". Information Technology & People.

[CR92] Macdonald JR, Corsi TM (2013). Supply chain disruption management: Severe events, recovery, and performance. Journal of Business Logistics.

[CR93] Martin, R. A. (2020). Visibility & Control: Addressing Supply Chain Challenges to Trustworthy Software-Enabled Things. In 2020 IEEE Systems Security Symposium (SSS) (pp. 1–4). IEEE

[CR94] Meisel NA, Williams CB, Ellis KP, Taylor D (2016). "Decision support for additive manufacturing deployment in remote or austere environments". Journal of Manufacturing Technology Management.

[CR95] Mishra D, Gunasekaran A, Papadopoulos T, Childe SJ (2018). Big Data and supply chain management: a review and bibliometric analysis. Annals of Operations Research.

[CR96] Moktadir MA, Ali SM, Paul SK, Shukla N (2019). Barriers to big data analytics in manufacturing supply chains: A case study from Bangladesh. Computers & Industrial Engineering.

[CR97] Mubarik MS, Naghavi N, Mubarik M, Kusi-Sarpong S, Khan SA, Zaman SI, Kazmi SHA (2021). Resilience and cleaner production in industry 4.0: Role of supply chain mapping and visibility. Journal of Cleaner Production.

[CR98] Min H (2019). Blockchain technology for enhancing supply chain resilience. Business Horizons.

[CR99] Munim ZH, Dushenko M, Jimenez VJ, Shakil MH, Imset M (2020). Big data and artificial intelligence in the maritime industry: a bibliometric review and future research directions. Maritime Policy & Management.

[CR101] Naghshineh, B., & Carvalho, H. (2020). The Impact of Additive Manufacturing on Supply Chain Resilience. In Doctoral Conference on Computing, Electrical and Industrial Systems (pp. 214–221). Springer, Cham

[CR102] Nah, F. F. H., & Siau, K. (2020, July). COVID-19 pandemic–role of technology in transforming business to the new normal. In International Conference on Human-Computer Interaction (pp. 585–600). Springer, Cham

[CR103] Papadopoulos T, Gunasekaran A, Dubey R, Altay N, Childe SJ, Fosso-Wamba S (2017). The role of Big Data in explaining disaster resilience in supply chains for sustainability. Journal of Cleaner Production.

[CR104] Park, K. T., Son, Y. H., & Noh, S. D. (2020). The architectural framework of a cyber physical logistics system for digital-twin-based supply chain control.International Journal of Production Research,1–22

[CR105] Paul J, Criado AR (2020). The art of writing literature review: What do we know and what do we need to know?. International Business Review.

[CR106] Pramanik D, Mondal SC, Haldar A (2020). "Resilient supplier selection to mitigate uncertainty: soft-computing approach". Journal of Modelling in Management.

[CR107] Queiroz MM, Wamba SF (2019). Blockchain adoption challenges in supply chain: An empirical investigation of the main drivers in India and the USA. International Journal of Information Management.

[CR108] Queiroz MM, Telles R, Bonilla SH (2020). "Blockchain and supply chain management integration: a systematic review of the literature". Supply Chain Management.

[CR109] Rajesh R (2020). A grey-layered ANP based decision support model for analyzing strategies of resilience in electronic supply chains. Engineering Applications of Artificial Intelligence.

[CR110] Ralston P, Blackhurst J (2020). Industry 4.0 and resilience in the supply chain: a driver of capability enhancement or capability loss?. International Journal of Production Research.

[CR111] Ramirez-Peña M, Sotano AJS, Pérez-Fernandez V, Abad FJ, Batista M (2020). Achieving a sustainable shipbuilding supply chain under I4. 0 perspective. Journal of Cleaner Production.

[CR112] Raut RD, Mangla SK, Narwane VS, Dora M, Liu M (2021). Big Data Analytics as a mediator in Lean, Agile, Resilient, and Green (LARG) practices effects on sustainable supply chains. Transportation Research Part E: Logistics and Transportation Review.

[CR113] Rejeb A, Simske S, Rejeb K, Treiblmaier H, Zailani S (2020). Internet of Things research in supply chain management and logistics: A bibliometric analysis. Internet of Things.

[CR114] Reyes PM, Visich JK, Jaska P (2020). Managing the dynamics of new technologies in the global supply chain. IEEE Engineering Management Review.

[CR115] Rialp A, Merigó JM, Cancino CA, Urbano D (2019). Twenty-five years (1992–2016) of the International Business Review: A bibliometric overview. International Business Review.

[CR116] Rialti, R., Marzi, G., Ciappei, C., & Busso, D. (2019). “Big data and dynamic capabilities: a bibliometric analysis and systematic literature review”,Management Decision, 57 No. 8,2052–2068

[CR117] Rice JB, Caniato F (2003). Building a secure and resilient supply network. Supply Chain Management Review.

[CR118] Rindfleisch A, Heide JB (1997). Transaction cost analysis: Past, present, and future applications. Journal of marketing.

[CR119] Rodríguez-Espíndola O, Chowdhury S, Beltagui A, Albores P (2020). The potential of emergent disruptive technologies for humanitarian supply chains: the integration of blockchain, Artificial Intelligence and 3D printing. International Journal of Production Research.

[CR121] Salmi M, Akmal JS, Pei E, Wolff J, Jaribion A, Khajavi SH (2020). 3D printing in COVID-19: productivity estimation of the most promising open source solutions in emergency situations. Applied Sciences.

[CR122] Shahzad A, Zhang K, Gherbi A (2020). Intuitive development to examine collaborative iot supply chain system underlying privacy and security levels and perspective powering through proactive blockchain. Sensors.

[CR123] Sharma, R., Shishodia, A., Kamble, S., Gunasekaran, A., & Belhadi, A. (2020). Agriculture supply chain risks and COVID-19: mitigation strategies and implications for the practitioners.International Journal of Logistics Research and Applications,1–27

[CR124] Singh NP, Singh S (2019). Building supply chain risk resilience: Role of big data analytics in supply chain disruption mitigation. Benchmarking: An International Journal.

[CR125] Strozzi F, Colicchia C, Creazza A, Noè C (2017). Literature review on the ‘Smart Factory’concept using bibliometric tools. International Journal of Production Research.

[CR126] Swift C, Guide Jr VDR, Muthulingam S (2019). Does supply chain visibility affect operating performance? Evidence from conflict minerals disclosures. Journal of Operations Management.

[CR127] Tang CS, Veelenturf LP (2019). The strategic role of logistics in the industry 4.0 era. Transportation Research Part E: Logistics and Transportation Review.

[CR128] Tao D, Wang T, Wang T, Zhang T, Zhang X, Qu X (2020). A systematic review and meta-analysis of user acceptance of consumer-oriented health information technologies. Computers in Human Behavior.

[CR129] Van Eck NJ, Waltman L (2010). Software survey: VOSviewer, a computer program for bibliometric mapping. Scientometrics.

[CR130] Van der Elst LA, Gokce Kurtoglu M, Leffel T, Zheng M, Gumennik A (2020). Rapid fabrication of sterile medical nasopharyngeal swabs by stereolithography for widespread testing in a pandemic. Advanced engineering materials.

[CR131] Verboeket V, Krikke H (2019). The disruptive impact of additive manufacturing on supply chains: A literature study, conceptual framework and research agenda. Computers in Industry.

[CR132] Wamba, S. F., & Queiroz, M. M. (2020). *Industry 4.0 and the supply chain digitalisation: a blockchain diffusion perspective* (pp. 1–18). Production Planning & Control

[CR133] Wang, Y., Chen, C. H., & Zghari-Sales, A. (2020). Designing a blockchain enabled supply chain.International Journal of Production Research,1–26

[CR134] Wang Y, Singgih M, Wang J, Rit M (2019). Making sense of blockchain technology: How will it transform supply chains?. International Journal of Production Economics.

[CR135] Witkowski K (2017). Internet of things, big data, industry 4.0–innovative solutions in logistics and supply chains management. Procedia engineering.

[CR136] Wong CY (2021). "Editorial". International Journal of Physical Distribution & Logistics Management.

[CR137] Xu LD, Xu EL, Li L (2018). Industry 4.0: state of the art and future trends. International Journal of Production Research.

[CR138] Xu S, Zhang X, Feng L, Yang W (2020). Disruption risks in supply chain management: a literature review based on bibliometric analysis. International Journal of Production Research.

[CR139] Yan B, Chen X, Yuan Q, Zhou X (2020). Sustainability in fresh agricultural product supply chain based on radio frequency identification under an emergency. Central European Journal of Operations Research.

[CR140] Yu W, Zhao G, Liu Q, Song Y (2021). Role of big data analytics capability in developing integrated hospital supply chains and operational flexibility: An organizational information processing theory perspective. Technological Forecasting and Social Change.

[CR141] Zavala-Alcívar A, Verdecho MJ, Alfaro-Saíz JJ (2020). A conceptual framework to manage resilience and increase sustainability in the supply chain. Sustainability.

[CR142] Zhang X, Yu Y, Zhang N (2020). "Sustainable supply chain management under big data: a bibliometric analysis". Journal of Enterprise Information Management.

[CR143] Zhao K, Yu X (2011). A case based reasoning approach on supplier selection in petroleum enterprises. Expert Systems with Applications.

[CR144] Zouari D, Ruel S, Viale L (2021). "Does digitalising the supply chain contribute to its resilience?“. International Journal of Physical Distribution & Logistics Management.

